# Associations between the neighbourhood social environment and preschool children’s physical activity and screen time

**DOI:** 10.1186/s12889-022-13493-2

**Published:** 2022-05-28

**Authors:** Jessica Baldwin, Lauren Arundell, Jill A. Hnatiuk

**Affiliations:** 1 School of Psychology, Deakin University, Geelong, Australia; 2 Institute for Physical Activity and Nutrition (IPAN), School of Exercise and Nutrition Sciences, Deakin University, Geelong, Australia

**Keywords:** Preschool-aged children, Neighbourhood, Social environment, Physical activity, Screen time, Movement guidelines

## Abstract

**Background:**

The neighbourhood social environment (NSE) has been associated with physical activity and screen time behaviours in adults and youth however less is known about this relationship in preschool-aged children (2–5 years). This study seeks to explore associations between the NSE and the physical activity and screen time behaviours of preschool-aged children.

**Method:**

Cross-sectional data was collected in 2019. Parents *(n* = 214) of preschool-aged children (*m* = 3.8 ± 0.8 years), from 187 different Australian postcodes representing all states and territories were invited to complete an online survey where they answered questions about their NSE (perceived social cohesion, social interaction, sense of community, social norms and neighbourhood crime) and proxy-reported their child’s usual physical activity and screen time (minutes/day). Two hierarchical linear regressions were run separately to assess relationships between NSE predictor variables and physical activity and screen time. Three logistic regressions were run to determine associations between NSE constructs and the likelihood of meeting: 1) physical activity (≥ 180 min/day including ≥ 60 min of moderate-to-vigorous-intensity), 2) screen time (≤ 60 min/day) and 3) both physical activity and screen time guidelines. Child age, gender, childcare attendance, and neighbourhood level socioeconomic status (SES) were controlled for in all analyses.

**Results:**

Social interaction was associated with increased daily physical activity (*b* = 17.76, 95%CI = 0.81, 34.71), decreased daily screen time (*b* = -12.77, 95%CI = -23.23, -2.23) and improved the likelihood of meeting physical activity (OR = 1.81, 95%CI = 1.20, 2.75) and combined physical activity and screen time guidelines (OR = 1.51, 95%CI = 1.03, 2.21). Higher neighbourhood crime was associated with a lower likelihood of meeting screen time guidelines (OR = 0.47, 95%CI = 0.47, 0.99). Social cohesion, sense of community and social norms were not statistically significant predictors of daily physical activity, screen time or meeting guidelines.

**Conclusion:**

Social interaction showed the most consistent associations with physical activity and screen time. Future research should consider potential mediators of this relationship, including parental facilitation of children’s outdoor time. Improving understanding of the relationship between the NSE and physical activity and screen time in young children can help to guide community-based initiatives striving to optimise behavioural, health and social outcomes.

## Background

Preschool-aged children (2–5 years) should engage in at least 180-min of physical activity, including 60-min of moderate-to-vigorous-intensity physical activity (MVPA), and no more than 60-min of sedentary screen time per day [1]. Globally, adherence to these movement guidelines is low [2], with only 16.9% of Australian preschool aged children meeting both the Australian physical activity (93.1% meeting) and screen time (17.3%) guidelines in a recent study [3]. Being active during the preschool years is essential for motor-skill development [4], maintaining a healthy weight [5] and developing problem-solving skills [6]. Excessive screen time is associated with increased adiposity, poor motor and social skill development [7] and language and literacy skill delays [8]. Physical activity has been found to be negatively associated with screen time in young children (3–7 years) over time [9], and both behaviours have been shown to track throughout childhood and beyond [10, 11], suggesting the earlier healthy habits are established the better.

The ecological model posits that a range of individual, social, environmental and policy factors influence children’s physical activity and screen time [12]. A 2016 systematic review synthesising correlates and determinants of young children’s (0–6 years) physical activity found that within the social domain specifically, numerous parent related factors had been investigated, but very little focus into the broader neighbourhood social environment (NSE) had occurred [13]. Whilst an array of terms have been used to describe aspects of the NSE [14], overall a positive NSE is characterised by a strong sense of connectedness, belonging, trust and safety, while negative NSE’s are more likely to lack cohesion and have a higher crime rate [14].

The NSE has been found to influence health behaviours in adults [14] and youth [15, 16], however research on younger children is extremely limited [14]. Australian findings have suggested parental perceived social cohesion, sense of community and social norms for walking is associated with higher physical activity levels (at least 3 h of active play on at least 5 days per week) in preschool-aged children who reside within 5 km of a park [17]. Qualitative findings have supported this with parents of 2–4 year old’s reporting that improved social support increases co-participation in physical activity while social views that differ from their own (norms) may act as a barrier [18]. This aligns with international findings in older children [19], and research examining preschool- aged children’s outdoor play [20, 21]; a known correlate of physical activity [20]. Conversely, neighbourhood crime has been associated with reduced physical activity in school aged children [20, 22] but only one study has been conducted in preschool aged children, which found no association [21].

Evidence suggests the NSE can also influence children’s screen time, for example high social neighbourhood disorder has been associated with an increased risk of excessive screen time use in Canadian youth [22]. Findings are however limited, particularly among preschool children. Higher social cohesion, sense of community and norms for walking have been associated with reduced levels of screen time in preschool-aged children [17]. Regarding neighbourhood crime, parental perceptions of higher crime have been linked to increased screen time in preschool children [23] and primary-school aged children [15, 24]. It is possible that parents may encourage screen-based activities at this age to avoid exposure to the NSE [25] and for ease of supervision [24].In summary, despite some evidence suggesting that the NSE may be important for children's physical activity and screen time, there remains a paucity of evidence focused on preschool-aged children and across a range of NSE domains. Therefore, this study aims to examine associations between five constructs of the NSE and physical activity and screen time behaviours in Australian preschool-aged children (2–5 years). It is hypothesised that more positive NSEs (i.e., higher social cohesion, more social interactions, greater sense of community, aligned social norms and reduced neighbourhood crime) will independently predict higher levels of physical activity and lower levels of screen time. It is further hypothesised that positive NSEs will increase the likelihood of preschool-aged children achieving physical activity, screen time and combined (physical activity and screen time) guidelines.

## Method

### Participants

**Fig. 1:**
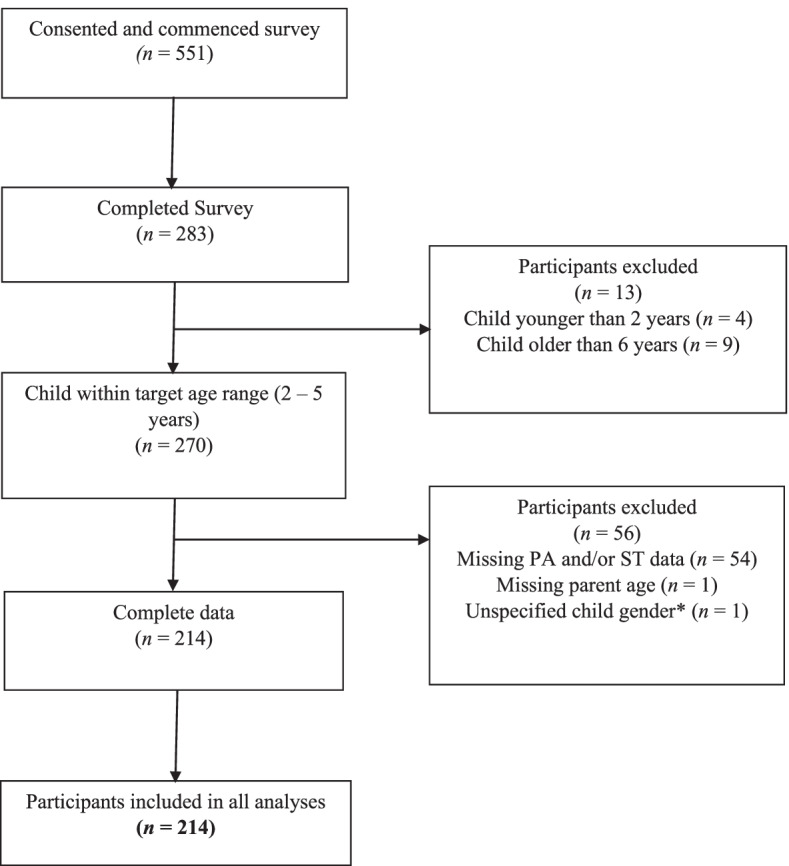
Participant Flow Chart

Ethical approval was received from the Deakin University Human Ethics Advisory Group (HEAG-H 47_2019) for the collection of cross-sectional data in 2019 through the SPACES (Screen time, Physical Activity in Children’s Environments Study) survey. Parents of preschool-aged children from across Australia were invited through online blogs and social media to complete the online survey (hosted by Qualtrics). The survey included questions relating to their perceptions of the NSE and proxy-reports of their child’s physical activity and screen time behaviours. A total of 669 parents provided informed consent however 118 did not provide any survey data. Figure 1 details participant eligibility and removal from a starting point of *n* = 551. Participants were not included in the analysis if they did not complete the survey (*n* = 268), their child was outside of the target age range (*n* = 13) or they did not provide complete outcome data for both physical activity and screen time (*n* = 56). A final sample of 214 participants with complete data was used for all analyses. Comparisons between the sample included in the analysis and those excluded for insufficient data showed that children included in the analyses reported slightly lower daily minutes of screen time than those excluded (98.1 vs 103.8, *P* < 0.05) however no other differences on child age, daily physical activity or meeting guidelines were found. No differences were found for parental age (t(257) = -0.58, *P* = 0.563). There was a small difference in parent sex with 99.5% of included parents being mothers and 100% of excluded parents being mothers. There was also a small group difference in the SES of included and excluded parents (t(222) -0.75, *P* = 0.429), with excluded participants being of a slightly higher SES.

The vast majority of participants completed the survey in June/July, hence seasonality was unlikely to be an issue in this sample.

### Measures

#### Neighbourhood social environment predictor variables

The constructs used in this study were guided by recommendations put forth by Kepper and colleagues [14]. The NSE elements are described below and individual survey items and scoring information for each of the NSE elements can be seen in Table 1.

**Table 1:** Neighbourhood Social Environment Predictor Variables and Outcome Variables

Scale Items	Response Options and Scoring
**Social Cohesion**	
People around my neighbourhood are willing to help their neighbours	5-point Likert scale (*Agree strongly, agree somewhat, neither agree nor disagree, disagree somewhat, disagree strongly)* Range 1–5
This is a close knit neighbourhood	
People in this neighbourhood can be trusted	
People in this neighbourhood generally don’t get along with each other (r)	
People in this neighbourhood do not share the same values (r)	
**Social Interaction**	
*In the last month, please indicate on how many days you did the following:*	4-point Likert scale *(Never, once or twice, two or three times, four or more times)* Range 1—4
Waved to a neighbour	
Said hello to a neighbour	
Stopped and talked with a neighbour	
**Sense of Community**	
My neighbourhood is a good place for my kids to grow up and thrive	5-point Likert scale *(Agree strongly, agree somewhat, neither agree nor disagree, disagree somewhat, disagree strongly)* Range 1 – 5
I expect to live in this neighbourhood for a long time	
This neighbourhood is a good place for me to live	
It is important for me to live in this particular neighbourhood	
I feel at home in this neighbourhood	
People in my neighbourhood share the same values	
I care about what my neighbours think of my actions	
I can recognise most of the people who live in my neighbourhood	
I have influence over what this neighbourhood is like	
If there is a problem in this neighbourhood, people who live here can get it solved	
People in this neighbourhood get along with each other	
My neighbours and I want the same things from this neighbourhood	
Very few of my neighbours know me (r)	
**Social Norms**	
I have similar views and practices to others in my neighbourhood regarding children's physical activity	5-point Likert scale *(Strongly disagree, disagree, neither agree nor disagree, agree, strongly agree)* Range 1 – 5
I have similar views and practices to others in my neighbourhood regarding children's screen time	
I have similar views and practices to my family/friends regarding children's physical activity	
I have similar views and practices to my family/friends regarding children's screen time	
**Neighbourhood Crime**	
There is a high crime rate in our neighbourhood	5-point Likert scale *(Strongly disagree, disagree, neither agree nor disagree, agree, strongly agree)* Range 1 – 5
There is a high presence of drug use in our neighbourhood	
There is a high prevalence of violence in our neighbourhood	
**Physical Activity**	
*On a usual day about how much time does your child spend doing the following activities on a usual day:*	Respond in hours and minutes
Physical activity that is highly energetic in nature (e.g. running, jumping, twirling etc.)	
Pottering (slow easy movements or standing play e.g. cooking and baking, water and sand play, dress ups etc.)	
**Screen Time**	
*On a usual day about how much time does your child spend doing the following activities on a usual day:*	Respond in hours and minutes
TV/DVD viewing/sreaming (on a traditional TV)	
Tablet/smart phone (eg. iPhone/iPad) use for games /apps	
Tablet/smart phone (e.g. iPhone/iPad) use for watching content (e.g. television shows, movies, Youtupe)	
Computer/internet use (excluding games)	
Computer/online games or a game player that hooks up to a TV (e.g. Playstation/Nintendo/X-Box)	
Handheld electronic games (e.g. Nintendo Switch, Gameboy/Nintendo DS)	

After reversing negatively worded items, items for each scale were added together then averaged to give participant scores. These averaged scores were used for the analyses. (r) – items were reverse coded prior

Social cohesion was measured using items adapted from Sampson et al. [26]. Participants were asked to rate their agreement on a five-point likert scale (strongly agree to strongly disagree) with five statements about cohesion within their neighbourhood. Items were reverse-scored to allow high scores to indicate high levels of social cohesion before averaging. Internal consistency for current sample was sufficient (Cronbach’s α = 0.79).

Social interaction (social relationships) was measured using the average of three items taken from a previously developed four-item scale which was shown to be valid and reliable (Cronbach’s α = 0.82) [27]. These items were intended to quantify (never, once or twice, two or three times, four or more times) the social interactions participants had with neighbours over the past month. Higher scores indicated more interactions. Internal consistency for current sample was good (Cronbach’s α = 0.89).

Sense of community (belonging) items asked participants to use a five-point likert scale (strongly agree to strongly disagree) to indicate their agreement with 13 items relating to how connected they felt within their neighbourhood. These items were drawn from Parker et al. (Cronbach’s α = 0.80) [27]. Items were reverse-scored to allow high scores to indicate a greater sense of community and low scores to indicate a lower sense of community prior to averaging. Internal consistency for current sample was sufficient (Cronbach’s α = 0.87).

Social norms items asked participants to use a five-point likert scale (strongly disagree to strongly agree) to indicate their agreement with four statements regarding views and practices specific to child physical activity and screen time. These items were designed based on previous qualitative work with mothers of pre-school aged children [18] hence reliability analyses was conducted. Internal consistency was adequate (Cronbach’s α = 0.70) and inter-rater reliability scores (kappa range = 0.251—0.451) were acceptable [28]. Item scores were averaged with higher scores indicating higher ratings of social norms.

Neighbourhood crime items asked participants to rate on a five-point likert scale (strongly disagree to strongly agree) their agreement with three statements relating to local crime. One of the items (“there is a high crime rate in my neighbourhood”) was taken from the Neighbourhood Environment Walkability Scales (NEWS) with a test–retest reliability of 0.71 [29]. Two items were designed and all three items were subjected to reliability analyses. Internal consistency was sufficient (Cronbach’s α = 0.88) and inter-rater reliability scores (kappa range = 0.483—0.568) were acceptable [28]. Item scores were averaged with higher scores indicating higher neighbourhood crime.

#### Child physical activity and screen time outcome measures

Parents were asked to proxy-report the amount of time (in hours and minutes) their child spent engaging in energetic play and pottering (slow easy movements) on a usual day over the past month. Energetic play was used to measure MVPA, and pottering was used to measure light-intensity physical activity (LPA). These items were based on previously developed items [30] used to measure young children’s physical activity which were shown to be valid and reliable measures (ICC range = 0.63—0.75) and adapted to match the current physical activity guidelines. Total physical activity was determined by adding MVPA and LPA time together. Parents also reported the amount of time (in hours and minutes) their child spent engaging in various forms of screen time as based on previously developed valid and reliable items [31] (ICC range = 0.51 – 0.69) on a usual day over the past month. Six items (TV, tablet viewing, tablet gaming, computer use, online games, handheld electronics) were combined to give total screen time. A further three binary variables were created to identify those meeting physical activity guidelines (dichotomised at ≥ 180 min of total physical activity including ≥ 60 min of MVPA), screen time guidelines (dichotomised at ≤ 60 min of screen time) and both guidelines (dichotomised at ≥ 180 min of physical activity including ≥ 60 min of MVPA and ≤ 60 min of screen time).

#### Sociodemographic covariates

Information on child age, gender (male, female, prefer not to specify) and childcare attendance was proxy-reported by the parent. For childcare attendance, a dichotomous variable was created in which children who attended either long daycare or family daycare for at least one day per week were classed as attending childcare while all others were classed as not attending childcare. The decision to dichotomise this variable was made given the substantial number of children not in care at all (35.3%), and to avoid having very small categories. Parent age, relationship to the subject child and residential postcode were self-reported. Postcodes were used to determine area-level socioeconomic status using the state-level suburb of residence Index of Relative Socioeconomic Advantage and Disadvantage decile [31]. Areas categorised in the higher deciles have greater relative levels of advantage and lower relative levels of disadvantage [31]. Quintiles were further created to represent five levels of SES [32]. Neighbourhood-level SES was used for this study given the specific focus on the neighbourhood social environment.

### Statistical analyses

IBM SPSS (Statistics Package for the Social Sciences) version 26 was used to run all statistical analyses. Physical activity and screen time variables were truncated at 2 standard deviations from the mean. This allowed high report times to be acknowledged and used without exerting undue influence over the results, similar to other studies [33]. For energetic play (MVPA) 13 cases were truncated to 285-min/day, for total physical activity 10 cases were truncated to 475-min/day, and for total screen time 6 cases were truncated to 245-min/day.

Prior to running the regression analyses, multicollinearity was checked and deemed unproblematic (VIF < 3). The continuous outcome variables were checked for normality (visual inspection of scatterplots) and the Durbin-Watson statistics fell within normal range (PA = 1.66, ST = 2.06). Two hierarchical linear regressions were conducted, one for each outcome variable (total PA minutes/day and total ST minutes/day). Covariates were controlled for by being entered at steps one (child age, gender, childcare attendance) and two (SES). NSE predictors were added at step three and model *R*^2^ *change* statistics were examined. Three binary logistic regressions were run to address the three binary outcome variables (meeting PA guidelines, meeting ST guidelines, meeting overall movement guidelines). Covariates were entered at step one followed by neighbourhood predictor variables at step two. Odds ratios of each predictor variable were examined.

## Results

A descriptive summary of the sample demographics can be found in Table 2. Participants were primarily mothers (99.5%) with an average age of 35.6 (± 4.1) years. The average child age was 3.8 (± 0.8) years and the majority were boys (57.7%). In this sample, 78% of children met physical activity guidelines, 42.5% met screen time guidelines and 34.6% met both physical activity and screen time guidelines.

**Table 2:** Sample Characteristics, *n* = 214

Characteristic	*%*	*Mean (SD)*	*Range*
Child Age		3.8 (1)	
*Child Gender*			
Male	57.7%		
Female	42.3%		
Attending childcare^a^	77.1%		
Parent Age		35.6 (4.1)	
*Parent Relationship to Child*			
Mother	99.5%		
Father	0.5%		
*Socioeconomic Status*^b^			
Highly Advantaged	29.9%		
Advantaged	26.0%		
Average	14.0%		
Disadvantaged	19.2%		
Highly Disadvantaged	11.2%		
PA^*d*^ (minutes/day)		257.6 (103.0)	
MVPA (minutes/day)		124.1 (73.1)	
ST (minutes/day)		98.1 (65.5)	
*Children Meeting Guidelines*			
Total PA (≥ 180 min/day)	82.7%		
MVPA (≥ 60 min/day)	87.9%		
Total PA including MVPA	78.0%		
ST (≤ 60 min/day)	42.5%		
PA, MVPA & ST	34.6%		
Social Cohesion		3.6 (0.7)	1—5
Social Interaction		3.0 (0.9)	1 – 4
Sense of Community		3.5 (0.6)	1 – 5
Social Norms		3.3 (0.6)	1 – 5
Neighbourhood Crime		2.2 (0.9)	1—5

^a^child attended long day care or family day care for at least one day per week

^b^calculated using postcode data

Overall, the final model of social cohesion, social interaction, sense of community, social norms and neighbourhood crime predicting children’s physical activity (Table 3) was not statistically significant (adjusted *R*^2^ = 0.02, *p* = 0.19). The NSE variables only predicted 3.7% (*R*^2^Change = 0.04, *p* = 1.597) of the variance in physical activity after controlling for covariates. Social interaction was the only significant individual predictor, accounting for 1.96% (sr^2^ = 0.02, *p* = 0.04) of the variance explained by the final model. Each one unit increase in parent-reported social interaction (on the four-point scale) was associated with an additional 17.76 (95% CI 0.81, 34.71) minutes of child daily physical activity.

**Table 3:** Hierarchical Linear Regression Results for Neighbourhood Social Environment Predicting Child PA and ST

Model/Predictor	B (95% CI)	SE B	β	R^2^	∆R^2^
Child PA				0.058	0.017
Social Cohesion	10.55 (-22.88, 41.99)	15.94	0.07		
Social Interaction	17.76* (0.81, 34.71)	8.60	0.16		
Sense of Community	-3.82 (-38.55, 30.9)	17.61	-0.02		
Social Norms	-17.87, (-43.04, 70.30)	12.77	-0.10		
Neighbourhood Crime	6.37 (-23.85, 11.1)	8.86	-0.05		
Child ST				0.113*	0.074
Social Cohesion	-10.59 (-29.98, 8.80)	9.83	-0.11		
Social Interaction	-12.77* (-23.23,—2.32)	5.30	-0.18		
Sense of Community	6.7 (-14.72, 28.12)	10.86	0.06		
Social Norms	6.73 (-8.80, 22.25)	7.87	0.06		
Neighbourhood Crime	7.94 (-2.84, 18.72)	5.47	0.10		

For each linear regression covariates were controlled for within models 1(child age, gender, childcare attendance) and 2 (SES). Values presented are from the final models [3]. These final models included social cohesion, social interactions, sense of community, social norms, neighbourhood crime, child age, gender, attending childcare and SES). Model 3 adjusted R^2^ change values were used to determine the overall significance of NSE predictors collectively after controlling for covariates

*B* unstandardised beta coefficients, *95%CI* 95% Confidence Interval, *β* standardised beta coefficient, *PA* Physical Activity, *ST* Screen Time

^*^significant at *p* < .05 level

For child screen time (Table 3), the overall model was statistically significant *(p* = 0.003), with NSE variables predicting 5.8% (R^2^ Change = 0.058) of the variance in screen time after controlling for covariates. Social interaction was again the only significant individual predictor in the final model, accounting for 2.56% (sr^2^ = 0.03, *p* = 0.017) of the variance explained. Each one unit decrease in parent-reported social interaction was associated with an additional 12.77 (95% CI -23.23, -2.32) minutes of child daily screen time.

Logistic regressions were run to determine whether NSE variables predicted the likelihood of preschool children meeting physical activity, screen time and both physical activity and screen time guidelines after controlling for covariates (Table 4). For physical activity the regression model was statistically significant, χ2(5) 11.28, *p* = 0.046, explaining 5% (R2LL = 0.05) of the variance in child physical activity and improving classification accuracy by 1% (from 78 to 79%) after controlling for covariates. Parental social interaction was the only significant predictor of children meeting the physical activity guidelines, with a one-unit increase improving the likelihood of meeting PA guidelines by 81.3%.

**Table 4:** Odds Ratios for Predictors of meeting PA, ST and Both PA/ST Guidelines

**Predictors**	***Model 1:*** ***Meeting PA (incl MVPA)***	***Model 2:*** ***Meeting ST***	***Model 3:*** ***Meeting Both PA & ST Guidelines***
	**OR (95% CI)**	**OR (95% CI)**	**OR (95% CI)**
Social Cohesion	0.97 (0.45, 2.09)	1.26 (0.65, 2.43)	1.37 (0.68, 2.75)
Social Interaction	1.81** (1.2, 2.75)	1.38 (0.96, 1.98)	1.51* (1.03, 2.21)
Sense of Community	1.08 (0.47, 2.49)	0.84 (0.40, 1.73)	1.01 (0.47, 2.17)
Social Norms	0.71 (0.37, 1.36)	0.90 (0.53, 1.54)	0.77 (0.44, 1.33)
Neighbourhood Crime	1.31 (0.84, 2.04)	0.47* (0.47, 0.99)	0.78 (0.53, 1.16)

^**^ significant at *p* < .01 level. * significant at *p* < .05 level. Meeting guidelines was dependant variable target category. Odds ratios calculated after controlling for child age, gender, attending childcare and SES in each model

*PA* Physical Activity, *MVPA* Moderate-to-vigorous- intensity Physical Activity, *ST* Screen Time, *CI* Confidence Interval

For screen time the logistic regression model was not significant χ2(5), 10.4, *p* = 0.065, explaining only 3.5% (R2LL = 0.04) of the variance in child screen time after controlling for covariates. Classification accuracy did not change from 60.7% when NSE predictors were added to the model. Crime was the only significant predictor, with a one unit increase in crime ratings (on the five-point scale), decreasing the likelihood of meeting screen time guidelines by 32%.

Logistic regression for meeting both physical activity and screen time guidelines was statistically significant χ2(5), 12.63, *p* = 0.027, with NSE predictors explaining 4.6% of the variance (R2LL = 0.046) and raising classification accuracy from 65% to 68.2% after controlling for covariates. Parental social interaction was the only significant predictor, with a one unit increase improving the likelihood of meeting both physical activity and screen time guidelines by 50.7%.

## Discussion

This study examined the relationship between a range of NSE constructs and preschool-aged children’s physical activity and screen time. There was little association between the NSE overall and child physical activity and screen time, however, parental social interaction and neighbourhood crime constructs were identified as being associated with these behaviours. Children whose parents reported having more frequent social interactions with neighbours were more likely to engage in higher amounts of physical activity, lower screen time and were more likely to meet the daily physical activity guideline, and both physical activity and screen time guidelines. Children whose parents reported higher levels of neighbourhood crime were more likely to exceed the daily screen time guidelines.

To our knowledge, this is the first study to quantitatively examine how social interaction, as a stand-alone dimension of the NSE, is associated with physical activity and screen time behaviours in children of any age. These findings are, however, aligned with previous research that found more frequent parental social interactions improve sense of social support for parents, which in turn is positively associated with children’s outdoor play [34]. Outdoor play has repeatedly been associated with increased physical activity and reduced screen time amongst children [20, 35] thus it may be that parental social interaction is related to child physical activity and screen time through children’s engagement in outdoor play [34]. For example, parents who spend more time outdoors with their children supervising or co-participating in physical activity have a greater chance of encountering and interacting with their neighbours [34]. Future research should examine parental facilitation of outdoor time as a mediator of social interaction and preschool children’s physical activity and screen time and utilise longitudinal or intervention designs to better understand these relationships. Community level strategies that endeavour to get young children outdoors may increase physical activity (and reduce screen time) whilst concurrently improving parental social interactions which will strengthen social support.

Higher neighbourhood crime was associated with a reduced likelihood of achieving screen time guidelines but was not linearly associated with the amount of screen time minutes per day. This suggests that efforts to reduce neighbourhood crime may not only improve overall perceived neighbourhood safety [36], but also improve screen time behaviours amongst young children. Previous research has found low neighbourhood safety (characterised by high crime) is linked to excessive screen time (> 2-h) in 3-year-olds compared to those living in safer neighbourhoods [23]. Among older children the findings are mixed with one study finding crime to be associated with increased screen time in American fourth-grade students (m = 9.1-years) [24], while another study found no differences in children’s (m = 9.1 ± 0.4 years) proxy-reported screen time in low versus high crime areas [37]. Neighbourhood crime and preschool children’s screen time data is extremely limited making it difficult to draw conclusions as to why there was no linear relationship, yet crime was associated with not meeting screen time guidelines. It may be that dichotomising the sample into meeting/not meeting guidelines resulted in reduced sensitivity of the measure which produced a significant result. This highlights the importance of examining screen time in both ways and may indicate that the use of guidelines as a threshold is effective for categorising people and identifying where elements of the NSE may be influential. More research is needed that assess screen time (linearly and dichotomously) to better understand the association with neighbourhood crime.

Social cohesion, sense of community and social norms were not associated with child physical activity and screen time in this sample. This contradicts previous Australian research that found social cohesion, sense of community and social norms for walking to be positively associated with physical activity and negatively associated with screen time in preschool children (*m* = 3.8 ± 0.8-years) living in an urban setting [17]. Social cohesion has also been associated with increased outdoor play in Dutch preschool children (4–6 years) living in an urban setting [21]. The present study included participants that represented the full range of neighbourhood-level SES however was skewed towards higher advantage. This may mean the results are not generalisable to the wider population, particularly those areas of higher disadvantage. Previous research has also indicated that the NSE may become a stronger predictor of physical activity and screen time as children get older and autonomy and independent mobility increases [15, 19]. Future research should use longitudinal study designs to examine how the influence of the NSE changes as children grow and enable causal conclusions regarding physical activity and screen time behaviours to be drawn.

Regarding meeting guidelines, the most recently collected nationally representative data (parent-reported) on meeting physical activity and screen time guidelines is from 2011/2012 which showed only 17% of preschool-aged children (2–5 years) met both recommendations with approximately 61% meeting physical activity guidelines and 25% meeting screen time guidelines [38]. Comparisons to smaller studies conducted in Australian 2–5-year-old’s offer further insights. For example, Cliff and colleagues [3] found 16.9% met both physical activity and screen time guidelines (93.1% met physical activity and 17.3% met screen time) while McNeill [39] found 94.3% met physical activity guidelines and only 17.8% met screen time guidelines (17.4% met overall movement guidelines however this was inclusive of 89.9% also meeting sleep recommendations). Both these previous studies used accelerometers to measure physical activity with screen time being parent reported. It may be that physical activity was underreported by parents in this study given only 78% of children in this sample met this guideline. Previous accelerometer-derived and parent proxy-report findings do however align with our present findings whereby higher than recommended screen time was observed causing the likelihood of meeting both guidelines to be reduced.

Strengths of this study were the inclusion of participants from across Australia, from a range of socioeconomic areas, examination of physical activity and screen time behaviours both linearly and dichotomously and use of the Kepper Framework [14] to guide NSE assessment. Limitations were the use of cross-sectional data meaning causal conclusions could not be drawn, small sample size, and generalisability limits given participants were predominantly mothers. Additionally, quantifying physical activity and differentiating between energetic (MVPA) and less intense activity (LPA) at this age is difficult [40], thus future research should look to using objective outcome measures (e.g., accelerometers) to improve accuracy. Neighbourhood-level SES was also controlled for within the analyses due to the focus being on the NSE, however it may be useful for future research to investigate how family-level SES may also influence the relationship between neighbourhood-level SES and physical activity and screen behaviours, for example through moderation analyses. Ultimately, associations of the NSE and health behaviours is understudied in preschool-aged children and further exploration is needed. Findings from this study have provided an important addition to the literature and can be used as a platform for future research. The 17 and 12 min difference in physical activity and screen time respectively found in the current study is indicative of 11% and 12% of the daily recommended amounts and so highlight an important consideration when trying to target children’s movement behaviours.

## Conclusion

Parental social interaction was positively associated with preschool children’s daily physical activity, their likelihood of meeting physical activity guidelines, and both physical activity and screen time guidelines, and negatively associated with their daily screen time. Further research should explore the potential mediators (e.g., parental facilitation of outdoor time) of this relationship. Neighbourhood crime was also negatively associated with meeting screen time guidelines. No associations were found between social cohesion, sense of community and social norms and child physical activity and screen time. Longitudinal research in larger sample sizes that starts during the preschool years and continues throughout childhood is needed to assess potential changes in the relationship between children’s NSE, physical activity and screen time over time. Increasing understanding of how the NSE may be associated with physical activity and screen time behaviours may help to identify neighbourhoods where young children are more at risk of not meeting guidelines and aid in the development of community-based initiatives that are aimed at improving health and social outcomes in this population.

## Data Availability

The datasets generated and/or analysed during the current study are not publicly available due to ethics board requirements but are available from the corresponding author on reasonable request.

## Abbreviations

NSE: Neighbourhood social environment
MVPA: Moderate-to-vigorous- intensity physical activity
LPA: Light intensity physical activity
